# Transcriptomic Profiling of the Four Adenosine Receptors in Human Leukocytes of Heart Failure Patients

**DOI:** 10.1155/2013/569438

**Published:** 2013-07-08

**Authors:** Manuela Cabiati, Raffaele Caruso, Alessandro Verde, Laura Sabatino, Maria-Aurora Morales, Silvia Del Ry

**Affiliations:** ^1^Laboratory of Cardiovascular Biochemistry, CNR Institute of Clinical Physiology, Via Giuseppe Moruzzi 1, 56124 Pisa, Italy; ^2^CNR Institute of Clinical Physiology, 20162 Milan, Italy; ^3^Cardiovascular Department, Niguarda Ca' Granda Hospital, 20162 Milan, Italy

## Abstract

In this study the transcriptomic profiling of adenosine receptors (ARs) in human leukocytes of heart failure (HF) patients as a function of clinical severity, assessing the possible changes with respect to healthy subjects (*C*), was evaluated. Total RNA was extracted from leukocytes of *C* (*n* = 8) and of HF patients (NYHA I-II *n* = 9; NYHA III-IV *n* = 14) with a PAXgene Blood RNA Kit. An increase as a function of clinical severity was observed in each AR (A_1_R: *C* = 0.02 ± 0.009, NYHA I-II = 0.21 ± 0.09, NYHA III-IV = 3.6 ± 1.3, *P* = 0.03  *C* versus NYHA III-IV, *P* = 0.02 NYHA I-II versus NYHA III-IV; A_2a_R: *C* = 0.2 ± 0.05, NYHA I-II = 0.19 ± 0.04, NYHA III-IV = 1.32 ± 0.33, *P* = 0.005  *C* versus NYHA III-IV, *P* = 0.003 NYHA I-II versus NYHA III-IV; A_2b_R: *C* = 1.78 ± 0.36, NYHA I-II = 1.35 ± 0.29, NYHA III-IV = 4.07 ± 1.21, *P* = 0.03: NYHA I-II versus NYHA III-IV; A_3_R: *C* = 0.76 ± 0.21, NYHA I-II = 0.94 ± 0.19, NYHA III-IV = 3.14 ± 0.77, *P* = 0.01  *C* versus NYHA III-IV and NYHA I-II versus NYHA III-IV, resp.). The mRNA expression of the ectonucleoside triphosphate diphosphohydrolase (CD39) and the ecto-5′-nucleotidase (CD73) were also evaluated. They resulted up-regulated. These findings show that components of adenosine metabolism and signalling are altered to promote adenosine production and signalling in HF patients. Thus, HF may benefit from adenosine-based drug therapy after confirmation by clinical trials.

## 1. Introduction

Adenosine is a potent extracellular messenger produced in high concentrations under metabolically unfavorable conditions [1]. Adenosine restores tissue homeostasis through the interaction with its membrane receptors, acting as a retaliatory metabolite [1, 2].

In particular, adenosine receptors such as A_2a_R receptors can contribute to tissue repair by enhancing paracrine adaptive mechanisms, by improving engraftment of circulating progenitor cells, or by promoting proliferation and differentiation of bone marrow mesenchymal stem cells in vivo [3]. Moreover, the cells of the immune system express adenosine receptors and may be the basis of adenosine modulatory effects in an inflammatory environment.

The ability of adenosine to inhibit cardiac fibroblast proliferation and collagen synthesis may help attenuate cardiac remodelling and fibrosis of ischemic or failing hearts [3, 4], thus preserving cardiac tissue architecture. Adenosine also shows an endogenous calcium antagonist-like effect, reducing the effects of calcium overload in the failing heart [5–7].

Despite great advances in the field of pharmacological and nonpharmacological treatment, at present [8], the rising epidemic of heart failure (HF) is an enormous medical and societal burden, since it affects 1%-2% of the adult population in western countries and ≥10% of the elderly population [9]. Therefore, greater knowledge of the mechanisms underlying the process of HF in all its multifactorial aspects is strongly warranted, also on a molecular basis.

Based on previous studies showing that in cardiovascular disease human circulating blood cells may mirror the same abnormalities occurring in the heart [10], studies have been carried out in peripheral blood mononuclear cells (PBMC) [11, 12], and recently A_1_R, A_2a_R, A_2b_R, and A_3_R mRNA expression was evaluated in human whole blood of normal subjects [13]. The four adenosine receptor subtypes resulted simultaneously expressed in leukocytes of healthy adults [13], providing an important and useful starting point for future studies devoted to evaluating the expression of ARs in human diseases characterized by a marked inflammatory component.

The aim of this study was to evaluate the transcriptomic profiling of ARs in human leukocytes of HF patients as compared to healthy subjects and to assess whether this profiling is related to the clinical severity of the disease.

## 2. Materials and Methods

### 2.1. Blood Collection and RNA Extraction

The study conforms with the principles outlined in the Declaration of Helsinki approved in 1964. The study was approved by the local Ethical Committee, and all patients provided signed informed consent.

Human whole blood samples (2.5 mL) from eight healthy adults and from 23 HF patients (New York Heart Classification-patients, NYHA I-II: *n* = 9 and NYHA III-IV: *n* = 14) were collected into PAXgene Blood RNA system (DIALAB ITALIA Srl) tubes.

The PAXgene Blood RNA system is an innovative new methodology for the collection, storage, and transport of blood, which efficiently stabilizes intracellular RNA and allows preservation of the samples at −20 to −70°C, maintaining the same degree of purity and stability of fresh blood.

Blood samples were processed with PAXgene Blood RNA kit (Qiagen, Milan, Italy) to obtain total RNA.

In all cases, the integrity of total RNA was detected by electrophoresis on Gel-Star (Lonza, Germany) stained 1.5% Agarose (Bio-Rad, Milan, Italy) gel; total RNA purity and concentration were evaluated spectrophotometrically (NanoDrop, Celbio, Milan, Italy). The RNA samples were stored at −80°C.

In Table 1 the clinical and biochemical characteristics of HF patients are reported.

### 2.2. Reverse Transcription

A quantity of 0.5 *μ*g of total RNA obtained from each sample was reverse-transcribed with iScript cDNA Synthesis Kit (Bio-Rad, Milan, Italy) according to the manufacturer's instructions.

### 2.3. Primer Synthesis and Real-Time PCR

Specific primers for adenosine receptors were synthesized by Qiagen: A_1_R (Hs_ADORA1_2_SG Quantitect Primer assay), A_2a_R (Hs_ADORA2a_1_SG Quantitect Primer assay), A_2b_R (Hs_ADORA2b_1_SG Quantitect Primer assay), and A_3_R (Hs_ADORA3_2_SG Quantitect Primer assay), while the ectonucleoside triphosphate diphosphohydrolase (CD39) and the ecto-5′-nucleotidase (CD73) designated for the dephosphorylation of extracellular ATP to AMP were synthesized by sigma-Aldrich (Table 2). Following recent guidelines [14, 15], three (*TOP2b, YWHAZ*, and *SRP14*) candidate reference genes (Table 2), from among the most commonly cited in the literature and previously selected [16], were used to normalize mRNA expression data obtained by Real Time-PCR (RT-PCR) in human whole blood. Reaction conditions of reference gene primer pairs were optimized. For Quantitect Primer assays, manufacturer's Real-Time conditions were followed, and a gradient RT-PCR was conducted to assess the optimal annealing temperature, while a dilution curve was always generated to verify RT-PCR efficiency as also reported in a previous study [13].

RT-PCR reactions were performed in a 96-well CFX96 RT-PCR system (Bio-Rad). The reaction was carried out in a total volume of 25 *μ*L per reaction. Reaction mixture included 2 *μ*L of template cDNA, 1 *μ*M of each primer, 2x QuantiFast SYBR Green SuperMix (Bio-Rad), and sterile water. Amplification protocol started with 95°C for 3 min followed by 39 cycles at 95°C for 10 s and 60°C for 30 s. To assess product specificity, amplicons were checked by melting curve analysis. Melting curves were generated from 65°C to 95°C with increments of 0.3°C/cycle. An interrun calibrator was used to allow comparison of *C*_*t*_ values obtained in different runs. The mean standard deviation for *C*_*t*_ values was 0.09. All reactions were performed in duplicate.

## 3. Data Analysis

GeNorm software was used to define the most stably expressed gene set, as previously described [14, 15]. The geometric mean of the three most stably expressed genes (*TOP2b*, *YWHAZ*, and *SRP14*) was used for normalization of each gene mRNA expression in the samples [16]. The relative quantification was performed by the ΔΔ*C*_*t*_ method using iQ5 software (BioRad). Differences between more than two independent groups were analyzed by Fisher's test after ANOVA. Differences between two independent groups were assessed by un-paired *t*-test. Relations between variables were assessed by linear regression.

The results are expressed as mean ± SEM, and *P* value was considered significant when <0.05.

## 4. Results and Discussion

The amount and purity of RNA were evaluated by absorption wavelengths of 260 nm and 280 nm, and the absorption ratio (*A*_260_/*A*_280_ nm) of all preparations was between 1.9 and 2.1. All samples were subject to gel electrophoresis to prove the integrity of 18 and 28 S ribosomal RNAs. 

**Figure 1:**
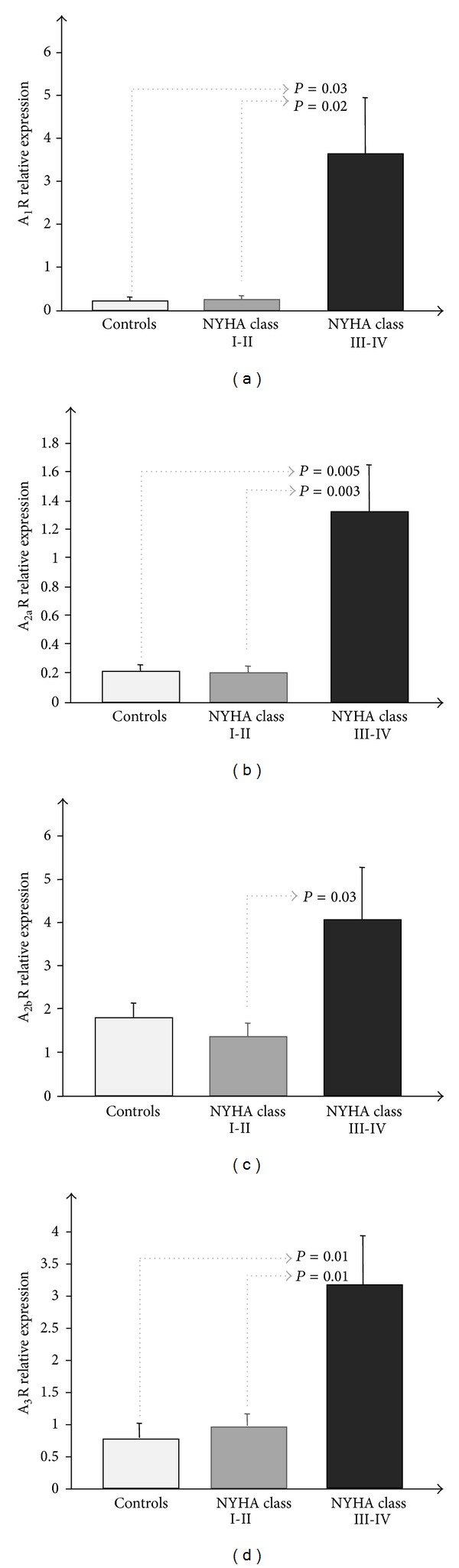
(a) A_1_R, (b) A_2a_R, (c) A_2b_R, and (d) A_3_R mRNA expression measured by Real-Time PCR in human whole blood of HF patients as a function of clinical severity.

In human whole blood of HF patients, a significant increase in the levels of mRNA expression of each AR as a function of clinical severity and compared to C was observed as reported in Figure 1.

To evaluate the catalyzation of the dephosphorylation of adenine nucleotides to adenosine in the extracellular space, the two cell surface molecules CD39 and CD73 were also evaluated. 

CD39 and CD73 transcript levels resulted upregulated in human leukocytes of HF patients as a function of clinical severity, although not significantly (CD39: *C* = 0.02 ± 0.009, NYHA I-II = 0.14 ± 0.04, NYHA III-IV = 0.13 ± 0.07; CD73: *C* = 0.15 ± 0.06, NYHA I-II = 0.25 ± 0.1, NYHA III-IV = 0.31 ± 0.07).

Significant correlations were observed between all ARs themselves (Table 3).

To our knowledge this is the first time that increased AR mRNA expression in the peripheral circulating cells of HF patients compared to healthy subjects has been demonstrated. The increased expression of these ARs appears related to the severity of the disease, as indicated by the progressive rise in mRNA expression values with worsening of symptoms, and which also parallels the degree of LV dilatation and dysfunction as shown by the impairment in LV ejection fraction.

In these patients all four receptor subtypes resulted activated despite their different functions at the cardiovascular level; these results emphasize possible adenosine receptor coregulation and are in line with a recent study which showed that normal cardiac homeostasis requires a stoichiometric balance between signaling through the A_1_/A_3_G*α*_i_ protein-coupled receptors and the A_2a_G*α*_s_ protein-coupled receptor. The need for “balanced” signaling provides an explanation for how a signaling system—in which a single ligand binds to multiple receptor subtypes—may develop naturally, although some of these active downstream pathways have diametrically opposite cellular effects [17].

As previously described [11], adenosine plasma levels could be increased in HF patients as a possible consequence of sympathetic activation, as also indirectly shown by CD39 and CD73 upregulation observed in these patients.

Adenosine is mainly present in the cytoplasm in its phosphorylated forms AMP, ADP, and ATP; it is generated through AMP hydrolysis by the CD73 enzyme that represents an integral part of energy regulation at cellular level.s

Under physiological conditions this nucleoside is kept to low concentration at the intracellular level by both S-adenosylhomocysteine and S-adenosylhomocysteine hydrolase [18].

In response to cellular stress and damage, such as metabolic factors, injury, and hypoxia, adenosine is generated in the extracellular space by dephosphorylation of adenine nucleotides released by cells [19], and this dephosphorylation is achieved by a two-step enzymatic process involving the CD39 (conversion of ATP/ADP to AMP) and CD73 (conversion of AMP to adenosine) [20].

Several neurohormonal factors, including catecholamines, renin angiotensin, and cytokines, are involved in the pathophysiology of chronic HF [21–23]. Activation of protein kinase C from either norepinephrine or angiotensin II activates CD73, and cytokines increase the transcriptional and protein levels of CD73 [24, 25] which may lead to increased plasma adenosine levels.

 The upregulation of these enzymes is an important purinergic remodeling response in environments where adenosine has been shown to regulate disease [26, 27].

The increased CD39 and CD73 transcript levels observed in this study, with a clear trend despite the lack of statistical significance, suggest a protective CD39-CD73-dependent adenosine production in advanced HF patients.

Adenosine attenuates the release of catecholamines, *β*-adrenoceptor-mediated myocardial hypercontraction, and calcium overload via A_1_ receptors. It also increases coronary blood flow and inhibits platelet and leukocyte activation via A_2_R. Furthermore, adenosine inhibits renin release and tumor necrosis factor-*α* production in experimental models [28, 29]. All these observations may lead to the hypothesis that increased adenosine levels in chronic HF may compensate for the worsening of the disease, suggesting that further elevation of adenosine levels may be beneficial to its pathophysiology. *β*-adrenoceptor blockers, angiotensin-converting enzyme inhibitors (ACE-Is), angiotensin receptor blockers, or aldosterone analogs have been proven effective for the treatment of HF in large-scale clinical trials [30, 31], but these strategies are not sufficient to effectively reduce mortality which remains high. Clinical observational studies showed a striking beneficial effect of endogenous adenosine accumulation achieved with oral chronic dipyridamole therapy on symptoms, exercise capacity, and left ventricular function in chronic HF [32, 33]. The hypothesis that increased endogenous adenosine accumulation via dipyridamole administration may exert a beneficial effect in HF is attractive; an ancillary anti-inflammatory effect on top of conventional anti-HF therapy would be desirable in an ideal “anti-heart failure” combination of drugs.

Moreover, an increasing body of evidence suggests an amplification of oxidative stress in circulating leukocytes of patients with HF, implying a contribution to the oxidative damage. Adenosine may have the potential to limit the oxidative stress response of leukocyte through an upregulation of A_2a_R [28, 29, 34] suggesting that HF patients might benefit from a pharmacologic manipulation of A_2a_R pathway in order to prevent exacerbations of cytotoxic leukocytes functions.

The results of this study are in line with previous ones performed in normal and failing minipig hearts [35, 36] and in peripheral blood mononuclear cells (PBMC) obtained by chronic heart failure patients [11, 12] but are in contrast with the study published by Asakura et al. [37] in which a downregulation of adenosine receptors was observed in the failing myocardium of chronic heart failure patients. The differences between the results of that study and ours remain unknown but may be attributable to the various degrees of severity of HF, the underlying causes of the disease, the normalization of RT-PCR results and a possible desensitization of adenosine receptors subtypes in presence of high adenosine concentrations [38, 39].

RT-PCR is the benchmark method for measuring mRNA expression levels, but the accuracy and reproducibility of its data greatly depend on appropriate normalization strategies. In order to avoid a confounding effect due to reference gene variability between samples, of the many strategies suggested [40, 41], the assessment of genes which are most stably expressed among a set of carefully selected targets [14] is considered the most reliable approach.

## 5. Conclusion

The findings of this study show that components of adenosine metabolism and signalling are altered to promote adenosine production in HF patients as a function of clinical severity. These changes include the upregulation of CD39-CD73 and elevations in each of the four AR subtypes. These data provide proof of the concept that adenosine-based drugs may play a role in the treatment of HF patients, after confirmation by randomized clinical trials.

## Figures and Tables

**Table 1:** Clinical and biochemical characteristics of heart failure patients.

	Patients NYHA I-II (*n* = 9)	Patients NYHA III-IV (*n* = 14)	*P*
Age, yrs	54 ± 4	53 ± 2	0.801
Male, *n* (%)	9 (100)	7 (50)	0.019
BMI, kg/m^2^	26 ± 3	26 ± 1	0.557
Diabetes, *n* (%)	1 (11)	3 (21)	1.000
Etiology, *n* (%)			0.611
IDC	8 (89)	10 (71)	
IHD	1 (11)	4 (29)	
LVEDV, mL	180 ± 33	268 ± 29	0.027
LVESV, mL	128 ± 29	202 ± 24	0.055
LVEF, %	33 ± 4	26 ± 2	0.159
MID, *n* (%)			0.074
0	1 (11)	—	
1+	5 (56)	2 (14)	
2+	2 (22)	8 (57)	
3+	1 (11)	4 (29)	
4+	—	—	
LA area, cm^2^	20 ± 2	39 ± 7	0.007
Therapy, *n* (%)			
ACEi	7 (79)	8 (57)	0.400
*β*-Blocker	9 (100)	14 (100)	1.000
Statin	4 (44)	7 (50)	1.000
Antiplatelets	6 (67)	8 (57)	1.000
Diuretic	7 (78)	14 (100)	0.142
C-reactive protein, mg/dL	0.3 ± 0.1	0.8 ± 0.3	0.410

Data are expressed as mean and SE or frequency (percentage).

BMI: body mass index; IDC: idiopathic dilated cardiomyopathy; IHD: ischemic heart disease; NYHA: New York Heart Classification; LVEDV: left ventricular end-diastolic volume; LVEF: left ventricular ejection fraction; LVESV: left ventricular end-systolic volume; MID: mitral insufficiency grade; LA: left atrium; ACEi: angiotensin converting enzyme inhibitor.

**Table 2:** Details of specific primers used in Real-Time PCR experiments.

Gene	Primer sequence	GenBank, accession number	Amplicon length, bp	Temperature, °C	Efficiency, %	*R* ^2^
*CD39 *	F: CACAGCATAGTAGATTGACAT	NM_001776	103	60	99.8	0.998
	R: ATACGCAGACAGAAGGAA					

*CD73 *	F: TAAGCACACTGTCTCATT	NM_002526	116	60	101	0.999
	R: CTGTATGGTCAAGTCAAC					

*YWHAZ *	F: ATGCAACCAACACATCCTATC	NM_001135702	178	60	95.3	0.997
	R: GCATTATTAGCGTGCTGTCTT					

*TOP2b *	F: AACTGGATGATGCTAATGATGCT	NM_001068	137	60	98.4	0.998
	R: TGGAAAAACTCCGTATCTGTCTC					

*SRP14 *	F: CAGATGGCTTATTCAAACCTCCT	NM_003134	181	60	99.9	0.998
	R: ATGCCCTTTACTGTGCTGCT					

*CD39: *ectonucleoside triphosphate diphosphohydrolase*; CD73: *ecto-5′-nucleotidase*; YWHAZ*: tyrosine 3-monooxygenase/tryptophan 5-monooxygenase activation protein z polypeptide; *TOP2b*: topoisomerase II Beta; *SRP14*: signal recognition particle 14 kDa.

**Table 3:** 

	*r*	*P*
A_1_R/A_2a_R	0.69	<0.0001
A_1_R/A_2b_R	0.51	0.008
A_1_R/A_3_R	0.65	0.0001
A_2a_R/A_2b_R	0.84	<0.0001
A_2a_R/A_3_R	0.83	<0.0001
A_2b_R/A_3_R	0.75	<0.0001
